# Disordered eating in office workers is correlated with trauma, stress, emotional regulation, and sleep quality: a cross-sectional screening

**DOI:** 10.3934/publichealth.2026024

**Published:** 2026-04-10

**Authors:** Nicola Magnavita, Lucia Isolani, Francesco Chirico, Sergio Garbarino, Martina Gasbarri, Egeria Scoditti, Loreta Di Michele

**Affiliations:** 1 Department of Security and Bioethics, Section of Occupational Health, Università Cattolica del Sacro Cuore, Rome 00168, Italy; 2 Occupational Health and Safety Unit, Public Health Department, AST Macerata, Macerata 62100, Italy; 3 Health Service Department, Italian State Police, Ministry of the Interior, Milan 00185, Italy; 4 Department of Neuroscience, Rehabilitation, Ophthalmology, Genetics and Maternal/Child Sciences (DINOGMI), University of Genoa, Genoa 16132, Italy; 5 Occupational Health, Local Sanitary Unit Roma4, Civitavecchia 00053, Italy; 6 National Research Council (CNR), Institute of Clinical Physiology (IFC), Lecce 73100, Italy; 7 Pulmonary Day Hospital and Interstitial Lung Diseases, San Camillo Forlanini Hospital, Rome 00152, Italy

**Keywords:** health surveillance, health promotion, anxiety, depression, obstructive sleep apnea, workplace, binge eating, obesity, driving accidents, lifestyle

## Abstract

Eating and feeding disorders (EDs) are a heterogeneous group of pathologies accompanied by important metabolic and psychiatric comorbidities that occur most frequently among the young but also appear in adulthood. Consequently, they can have a high impact on the quality of life and productivity. In the workplace, they are associated with traumatic events, stress, and sleep problems. Office workers who are not engaged in night shifts are an ideal sample for studying the relationship between sleep problems and EDs, since the confounding factor of night work can be excluded. This study is a survey of office workers undergoing health surveillance over a one-year period. Participants (463 out of a total of 489) completed the Short Version of the Eating Disorder Examination Questionnaire (EDE-QS) to screen for EDs, the Effort/Reward Imbalance (ERI) questionnaire for stress, the Goldberg's Anxiety and Depression Scale (GADS) for mental health, the Pittsburgh Sleep Quality Index (PSQI) for sleep quality, two items from the Stop-Bang questionnaire to screen for obstructive sleep apnea (OSA), and some questions concerning trauma, violence, motor vehicle accidents, and injuries. The prevalence of suspected EDs was 6% (CI 95% 4.1; 8.6). According to the body mass index, all suspected cases were affected by overeating. On the basis of non-smoking, regular physical activity, and adherence to appropriate alcohol and eating behaviors, only 1.9% of the cohort was classified as having a healthy lifestyle. Suspected EDs reported experiencing significantly more life trauma (OR = 4.47, CI 95% 1.99; 10.04), domestic injuries (OR = 3.89, CI 95% 1.04; 14.56), assaults (OR = 4.36, CI 95% 1.35; 14.07), and near-miss driving accidents (OR = 3.32, CI 95% 1.26; 8.76) than their colleagues in the previous year. They also reported higher levels of work-related stress than their colleagues (OR 5.38, CI 95% 2.29; 12.63), worse sleep quality (OR = 1.31, CI 95% 1.18; 1.46), and higher levels of anxiety (OR = 1.41, CI 95% 1.23; 1.62) and depression (OR = 1.55, CI 95% 1.33; 1.81). OSA symptoms were present in 25% of suspected EDs. A multivariate logistic regression model indicated that trauma and depression were most strongly associated with suspected ED cases. Health promotion in office workers should be based on sleep hygiene, stress reduction, and emotional trauma counseling.

## 1. Introduction

Eating and feeding disorders (EDs) consist of a heterogeneous group of conditions characterized by persistent modifications in eating behaviour [1],[2] and are often associated with psychiatric and medical complications [3]. Although the WHO International Classification of Diseases and Related Health Problems (ICD-11) [4] and the Diagnostic and Statistical Manual of Mental Disorders text, version 5 (DSM-5-TR) [5],[6], use different diagnostic criteria, they recognize as EDs not only conditions that result in a reduced food intake, but also others that, on the contrary, are characterized by overeating and commonly associated with obesity. Among the former, one of the most common, especially in young women, is anorexia nervosa (AN); among the latter, the most common are binge eating disorder (BED) and bulimia nervosa (BN). The two diagnostic criteria, ICD-11 and DSM-5, have convergent validity, but the former is more extensive, although not overly inclusive [7],[8]. The estimated prevalence of EDs varies depending on the criteria used. In the USA, data from the 2012–2013 National Epidemiologic Survey on Alcohol and Related Conditions based on DSM-5 criteria enabled Udo & Grilo [9] to estimate the 12-month prevalence of the most common forms of EDs in adults as 0.05% (SE 0.02%) for AN, 0.14% (SE 0.02%) for BN, and 0.44% (SE 0.04%) for BED. On the other hand, Qian et al. [10], who collected studies based predominantly on the ICD classification, estimated a 12-month prevalence of EDs to be 0.72%. In a narrative review, Keski-Rahkonen & Mustelin [11] observed that in Europe, <1%–4% of women reported AN, <1%–2% BN, 1%–4% BED, and 2%–3% OSFED, while 0.3%–0.7% of men reported EDs. In a systematic review of studies published over the 2000–2018 period, Galmiche et al [12] estimated a weighted point prevalence of 5.7% (0.9%–13.5%) for women and 2.2% (0.2%–7.3%) for men. These disorders are severely underdiagnosed: it is believed that only one in three cases receives a medical diagnosis [11].

EDs are most prevalent in adolescence and early adulthood [13]. They can persist in early adulthood in 40.7% of cases, with a relapse percentage of 24.5% [14], although in some cases they may appear for the first time in adulthood. Clusters of first-onset cases have been reported in polycystic ovary syndrome [15], in the perimenopausal period [16], and after cancer treatment [17]. Even loneliness, which is more frequent in older subjects [18], and negative interpersonal relationships, which include both real experiences and individuals' skewed perceptions, exacerbate eating disorders [19]. There is a noticeable prevalence of EDs among individuals of working age [20]. Since EDs are associated with serious physical health problems such as obesity, diabetes, metabolic syndrome [21], alcohol and drug use [22], smoking [23],[24], lack of exercise [25], and psychiatric disorders (especially post-traumatic stress disorder and depression) [26]–[33], they have a significant impact on work capacity and productivity. Some factors that may contribute to their onset, such as traumas, night work, and stress, are frequently encountered in the workplace. Consequently, ED screening should be included in the periodic health and safety checks carried out on workers by occupational health physicians.

The few studies conducted on EDs in the workplace indicate that certain occupational factors may play a role in the genesis of the disorders. These include night work [34],[35], which has been associated with EDs for many reasons, including potential chronic circadian disruption and sleep deprivation [36], atypical light exposure [37], and nocturnal eating [38], which may influence gut function, eating behavior, and microbiome interactions [39],[40]. However, to date, there are no longitudinal studies available that demonstrate these effects, and the association between night work and EDs has not always been confirmed by cross-sectional studies [20]. On the contrary, the association between sleep problems and binge eating [41] or anorexia [42] is well consolidated. Recently, it has been hypothesized that the orexin system is the neurobiological link of a reciprocal relationship between BED, which promotes EDs, and dysregulated sleep/wake patterns, which promote addictive eating [43]–[45]. According to the state of knowledge, evidence regarding the longitudinal relationship between eating habits, nutritional aspects, sleep dimensions (including duration, timing, quality, and insomnia symptoms), and physical health indicators (such as anthropometric indices, fat percentage, and obesity risk) remains limited [46], and more studies are needed.

Another occupational factor found to be associated with EDs is work-related stress and the occupational trauma that causes it, such as injuries, traffic accidents, and violence. EDs have an increased incidence in workers with PTSD [47]–[50] and in adults who suffered childhood adversity and maltreatment [51],[52] or have experienced workplace violence [53] or bullying [54]. Epigenetic mechanisms may connect traumatic events and life stress to eating disorders [55]. An atypical reaction to interpersonal stress could be a contributing factor to eating disorders; however, only limited research has been carried out on this subject [56].

Office workers are the ideal population to study the relationship between sleep and EDs, because their activity excludes night work. Therefore, this population offers an opportunity to study the effects of sleep deprivation without interference from night work. Furthermore, like all other workers, they may be exposed to occupational stress and the traumas of work and life with their ensuing emotional consequences (anxiety, depression, sleep disturbances). To the best of our knowledge, no one has yet studied the distribution of ED symptoms in this setting. We therefore set out to study the prevalence of suspect EDs in office workers and to assess the relationship of EDs with individual and occupational factors. In particular, we studied the association of suspected cases of EDs detected at screening with poor lifestyles, family trauma, traffic accidents, injuries, work-related aggression, stress, anxiety, and depression, and attempted to clarify whether sleep quality and sleep disorders are associated with the risk of EDs even in workers who do not work night shifts.

## 2. Materials and methods

### 2.1. Population

In 2022, in all the companies supervised by the first author of this article, office workers were invited to participate in a health promotion program that included screening for eating problems. The employees belonged to 20 companies from the health, social, industrial, and commercial sectors. Cases found to be positive after screening were given the option of specialist examination and possible treatment on the National Health Service. The invitation to participate was extended to all employees (N = 489) who underwent the periodic medical examination that year, without any incentive or exclusion criteria. The participants signed an informed consent and agreed that the results of the survey would be forwarded in a collective, anonymous form to their employer, the prevention service, and the workers' representatives for possible preventive measures. Participation was not compulsory, but was very high (94.7%), as is customary for this type of initiative [57].

### 2.2. Questionnaire

Screening for EDs was performed with the Italian version [58] of the Eating Disorder Examination Questionnaire, short form, EDE-QS [59]. The questionnaire consisted of 12 questions. The answer was graded according to a Likert scale, ranging from 0 = not at all, or 0 days a week, to 3 = markedly, or 6–7 days a week. A score above 15 points was considered indicative of EDs [60]. Workers screened with the EDE-QS can be classified as suspected ED cases according to DSM 5 criteria. Furthermore, by taking body mass index (BMI) into account, it is possible to predict the type of disorder and distinguish between suspected AN, BN, and BED. In this study, Cronbach's alpha of the questionnaire was 0.859.

Workers were also asked about factors potentially associated with EDs. Different types of traumas occurring in the previous year were investigated using binary yes/no questions. Workplace violence was investigated, using the first questions from Arnetz's Violent Incident Form (VIF) [61] to ascertain whether, in the previous year, the respondents had suffered physical assault, threats, harassment, or forms of persistent and intrusive violence such as stalking.

Similarly, with binary questions, we investigated the occurrence of major life trauma in the previous year, such as bereavement, serious economic or family problems, being involved in a road accident while driving, being on the verge of having a road accident while driving, and experiencing an accident at work or a domestic accident. Single questions were used to investigate smoking habits, alcohol consumption, physical exercise, and attention to limiting salt, sugar, or fat in meals in the previous week. Responses were graded according to a scale of four points.

Sleep quality was assessed using the Italian version [62] of the Pittsburgh Sleep Questionnaire Index (PSQI) [63]. With this tool, a score above 5 indicates poor sleep quality. In this study, Cronbach's alpha for the PSQI was 0.766.

Poor sleep quality and sleep deprivation may be the result of social and occupational factors or medical disorders, the most common being obstructive sleep apnea (OSA). We investigated the risk of OSA by following the guidelines of the Italian Interdisciplinary Technical Committee for Sleepiness and Safety in OSAS Patients [64], which recommended, for large surveys, the adoption of two binary questions from the STOP-Bang questionnaire [65],[66] relating to snoring and obstructive apnea.

Occupational stress was assessed using the Siegrist effort/reward model that considers distress as a consequence of a disparity between the effort expended at work and the material or intangible rewards received. We used the Italian version [67] of the effort/reward imbalance (ERI) [68], short form [69], that consists of 3 questions for effort and 7 for reward. The ERI is calculated as the weighted ratio between effort and reward. An ERI score greater than one indicates a condition of distress. The reliability of the scale was 0.821 for effort and 0.742 for reward.

The Italian version [70] of the Goldberg scale (GADS) [71], consisting of 18 binary questions, was used to measure the risk of anxiety and depression. The reliability of the two scales in this study was 0.823 for anxiety and 0.800 for depression.

### 2.3. Statistics

Statistical analysis focused first on studying the distribution of the variables of interest. The Kolmogorov–Smirnov and Shapiro–Wilk tests were used to ascertain whether their distribution was normal and also to calculate the mean, median, and standard deviation. Evaluation of the prevalence of disorders was integrated with that of the degree of uncertainty in prevalence (95% confidence interval, CI 95%) calculated by means of the Clopper–Pearson exact binomial test [72]–[75]. Mean comparisons were made using the student's t-test for parametric variables and the Mann–Whitney–Wilcoxon U-test for nonparametric data. We used ANOVA to compare three or more groups. The association between proportions was studied using the Pearson chi-square test with Yates continuity correction for frequencies lower than 25 units and Fisher's exact test for frequencies lower than 5 units, while the association between potential risk factors and EDs was calculated using univariate logistic regression, with suspected ED status as the dependent variable and individual risk factors as the independent variable. This enabled us to calculate the odds ratio (OR) and 95% confidence interval (CI) for all variables of interest. Finally, after entering all potential predictors as independent variables in a multiple logistic regression model, we assessed which ones retained a significant association with the risk of EDs. Before performing this calculation, the absence of collinearity between predictor variables was checked by calculating the variance inflation factor (VIF). All calculations were performed using the 30.0 version of the IBM/SPSS statistical package (IBM Corp.: Armonk, NY, USA).

### 2.4. Ethics approval of research

The study was conducted in accordance with the Declaration of Helsinki and approved by the Ethics Committee of the Università Cattolica del Sacro Cuore, Rome, Italy, on March 3, 2022 (ID 4671). The workers signed an informed consent form and authorized the use of their data anonymously, including for scientific purposes, in their personal health document. The research was not funded, but was conducted as part of the health surveillance duties of the companies assigned to the first author of this article.

## 3. Results

The study participants were 463 office workers (male 226, 48.8%, female 237, 51.2%), with an average age of 46.3 ± 10.24 years (Table 1). Most of the offices (357 people, 77.1%) were located within the capital, and the remainder (106, 22.9%) in neighboring urban centers. The companies where the offices were located operated in the health and social care (22.7%), retail (67.6%), and industry sectors (9.7%).

An average score of 4.9 ± 5.5 points was obtained for the EDE-QS questionnaire in the entire sample. No significant difference was observed in the distribution of EDE scores between the different work sectors of the offices (ANOVA, *p* = 0.835), nor in relation to the location of the office (Student's t-test, *p* = 0.287). Twenty-eight employees had an EDE-QS score above 15, indicating suspected ED. The prevalence of individuals with suspected EDs was 6.0 (CI 95% 4.1; 8.6). The BMI of workers with suspected ED was on average 28.45 ± 5.75, significantly higher than that of their colleagues (24.6 ± 4.17; Student's t-test, p < 0.001). All suspected EDs had a BMI above 18.5. Since there were no underweight individuals among the workers with suspected ED, it was possible to rule out a suspicion of AN. According to the authors of the questionnaire [34], the combined assessment of the EDE-QS questionnaires and BMIs indicated that a specialist examination would probably find that office workers with an abnormal EDE-QS score had a higher probability of a diagnosis of BED or less probably of BN or other specified feeding and eating disorders (OSFEDs).

**Table 1.** Comparison of cases of suspected EDs (EDE-QS score >15) with their screening-negative counterparts.

Variable	Suspect EDs (N = 28)	Other employees (N = 435)	*p*
Female gender (n = 237)	17 (7.2%)	220 (92.8%)	0.298^a^
Male gender (n = 226)	11 (4.9%)	215 (95.1%)	
Age (mean ± s.d.)	46.86 ± 11.55	46.27 ± 10.16	0.768^b^
Resident in Rome (n = 357)	22 (78.6%)	335 (77.1%)	0.849^a^
BMI (mean ± s.d.)	28.45 ± 5.75	24.59 ± 4.17	<0.001^b^
Obese (n = 46)	6 (24.0%)	40 (10.4%)	0.038^a^
Physical exercise (mean rank)	230.7	252.5	0.386^c^
Dietary control (mean rank)	232.8	219.8	0.602^c^
Alcohol (mean rank)	231.9	225.5	0.783^c^
Tobacco smoke (mean rank)	233.9	202.9	0.206^c^
Life trauma (n = 66)	11 (39.3%)	55 (12.6%)	<0.001^a^
Work injury (n = 8)	0	8 (1.8%)	1^d^
Domestic accident (n = 16)	3 (10.7%)	13 (3.0%)	0.030^a^; 0.065^d^
Driving accident (n = 8)	0	8 (1.8%)	1^d^
Driving near-miss (n = 39)	6 (21.4%)	33 (7.6%)	0.027^a^
Workplace violence (n = 20)	4 (14.3%)	16 (3.7%)	0.028^a^; 0.027^d^
Loud snoring (n = 30)	7 (25.0%)	23 (5.3%)	<0.001^a^
Sleep apnea (n =19)	4 (14.3%)	15 (3.4%)	0.005^a^; 0.022^d^
ERI work stress (mean ± s.d.)	1.17 ± 0.61	0.88 ± 0.34	<0.001^b^
PSQI poor sleep quality (mean ± s.d.)	9.07 ± 4.63	5.52 ± 2.41	<0.001^b^
Anxiety	5.07 ± 3.38	2.39 ± 2.44	<0.001^b^
Depression	4.18 ± 2.82	1.49 ± 1.94	<0.001^b^

Notes: ^a^: Pearson's chi-square; ^b^ Student's t-test; ^c^ Mann–Whitney U test; ^d^ Fisher's exact test. ERI: Effort reward imbalance. PSQI: Pittsburg Sleep Quality Inventory.

In the sample we examined, 11.3% of the participants (CI 95% 8.4%; 14.8%) were obese (BMI > 30). The proportion of obese individuals in the group of suspected EDs was significantly higher than in the rest of the sample (24.0% vs. 10.4%, Pearson Chi-square and Fisher's exact test, p < 0.05).

The prevalence of EDs was slightly higher in females than in males (7.2% vs. 4.9%), but the difference was not significant (Pearson's chi-square, p = 0.298). Using logistic regression, females had a higher odds ratio than males for having EDs (OR = 1.510); however, the confidence interval (CI 95% = 0.691; 3.299) included the unit and was therefore not significant.

Age was also not significantly associated with the risk of eating disorders (OR = 1.006, CI 95% = 0.969; 1.044) (Table 2).

**Table 2.** Association of risk factors with suspected cases of EDs. Univariate logistic regression.

Variable	Odds ratio	Confidence interval 95%	*p*
Female gender	1.510	0.691; 3.299	0.301
Age	1.006	0.969; 1.044	0.767
Life trauma	4.471	1.990; 10.044	<0.0001
Near-miss driving accident	3.322	1.259; 8.764	0.015
Domestic injury	3.895	1.042; 14.562	0.043
Workplace violence	4.365	1.354; 14.067	0.014
Work-related distress	5.379	2.291; 12.631	<0.0001
Loud snoring	5.971	2.303; 15.484	<0.0001
Sleep apnea	4.667	1.438; 15.144	<0.0001
Poor sleep quality	1.315	1.186; 1.457	<0.0001
Anxiety	1.409	1.227; 1.619	<0.0001
Depression	1.555	1.335; 1.811	<0.0001

Only one in four (27.2%) of the office workers reported doing some form of exercise (at least 30 minutes in the gym, cycling, playing football, swimming, running, etc. during their free time) three or more times a week, while 31.5% did not do any physical exercise. Only one in five (20.3%) said they had managed to limit salt, sugar, or fat in their meals in the previous week, while 41.7% said they had done so only occasionally or never. More than half of the office workers (55.2%) reported regular consumption of alcoholic beverages, and 5.4% reported exceeding the recommended limit of 7 units of alcohol (an average glass of wine, a 33 cl bottle of beer, or a small glass of spirits) per week. Approximately one-third (32.4%) were current smokers, and a further 20.7% had smoked in the past and switched to e-cigarettes; less than half (46.9%) had never smoked. Only 1.9% of our sample had a healthy lifestyle, defined as non-smoking, regular physical activity, and adherence to appropriate alcohol and eating behaviors. We observed no differences in the various habits and lifestyles between the suspected cases of EDs and the other workers. Specifically, we did not observe significant differences in alcohol consumption (Mann–Whitney U Wilcoxon W, *p* = 0.783), tobacco smoking (*p* = 0.206), physical exercise (*p* = 0.386), and dietary control (*p* = 0.602) between suspected cases of EDs and their colleagues.

Sixty-six workers (14.3%) reported having experienced a major life trauma in the previous year. Trauma was significantly associated with possible EDs. Serious trauma was reported by 39.3% of suspected cases of EDs, but only by 12.6% of employees with normal eating behavior (Pearson chi-square, *p* < 0.001). The risk of suffering from EDs for those who had experienced life trauma in the year prior to the medical examination increased very significantly (OR = 4.471, CI 95% 1.990; 10.044, *p* < 0.001) when compared to the other workers.

Eight workers (1.7%) had had an accident at work in the previous year. The distribution of cases showed no differences in relation to eating disorders. Sixteen people (3.5%) had had an accident at home. The rate of domestic accidents was significantly higher (*p* < 0.05) in suspected ED cases (10.7%) than in the other office workers (3.0%), but above the significance level when Fisher's exact test was applied. Domestic injury was significantly associated with the risk of EDs (OR = 3.895, CI 95% 1.042; 14.562, *p* < 0.05).

Among those surveyed, 8 had been involved in a road accident while driving, and 39 (8.4%) had risked being involved in a driving accident in the previous year. The rate of road accidents did not differ between the two groups, but near misses occurred significantly more frequently among suspected cases of EDs than among other workers (21.4% vs. 7.6%, chi-square, *p* = 0.011, Yates's continuity correction, *p* = 0.027, Fisher's exact test, *p* = 0.023). Workers who reported near-miss driving accidents were at a high risk of EDs (OR = 3.322, CI 95% 1.259; 8.764, *p* < 0.05).

Twenty employees (4.3%) reported having experienced some form of violence at work. Among workers suspected of having EDs, 14.3% reported having experienced violence, compared to 3.7% in the rest of the group (chi-square with Yates's continuity correction, *p* = 0.028, Fisher's exact test, *p* = 0.027). Workplace violence was significantly associated with ED risk (OR = 4.365, CI 95% 1.354; 14.067, *p* < 0.05).

In our sample, 30 office workers (6.5%, CI 95% 4.4; 9.1) reported loud snoring and waking up suddenly or feeling suffocated while snoring, and 19 (4.1%, CI 95% 2.5;6.3) confirmed that their partner had told them they had sleep apnea. Both symptoms were predominantly present in individuals with suspected EDs. Heavy snoring was present in 25% of suspected ED cases and in 5.3% of the other workers (chi-square, *p* < 0.001). Sleep apnea, objectively observed by a partner, was reported by 14.3% of suspected cases of ED and 3.4% of other employees (chi-square, *p* < 0.005; Fisher's exact test, *p* = 0.022). Both heavy snorers and apneic workers were at high risk of EDs.

**Table 3.** Occupational predictors of EDs, multivariate logistic regression.

Variable	Odds ratio	Confidence interval 95%	*p*
Age	0.999	0.957; 1.043	0.966
Female gender	0.859	0.332; 2.223	0.754
Work-related distress	2.161	0.721; 6.479	0.169
Poor sleep quality	1.097	0.921; 1.306	0.300
Anxiety	0.942	0.707; 1.255	0.684
Depression	1.436	1.095; 1.883	0.009
Workplace violence	0.599	0.118; 3.047	0.537
Domestic injury	0.823	0.126; 5.356	0.838
Life trauma	2.689	1.024; 7.061	0.045
Near-miss driving accident	0.705	0.150; 3.309	0.658
Loud snoring	1.282	0.368; 4.463	0.697
Sleep apnea	2.870	0.597; 13.785	0.188
Constant	0.001		0.000

Poor sleep quality was significantly associated with the risk of EDs (OR = 1.315, CI 95% 1.186; 1.457, *p* < 0.0001).

There was also a highly significant association between anxiety and depression and EDs. Anxiety had an OR = 1.409 (CI 95% 1.227; 1.619, *p* < 0.0001), while depression had an OR = 1.555 (CI 95% 1.335;1.811, *p* < 0.0001).

We entered all the above variables into a multivariate logistic regression model to test which had the closest relationship with the risk of EDs. Life trauma and depression were found to be the most significant predictors of EDs (Table 3).

## 4. Discussion

This study revealed that a significant number of office workers (6.0%, CI 95% 4.1; 8.6%) were suffering from suspected eating disorders. Considering that the EDE-QS questionnaire has high sensitivity (0.83) and specificity (0.85) and a positive predictive value of 0.37 [60], the estimated prevalence of clinically confirmed EDs in this sample could be higher than 2.2%. This prevalence is high compared to the general population, as shown by currently available meta-analysis studies [10]–[13] in which less than 1%–2% of adults are affected. This result contradicts the healthy worker effect, which suggests that workers enjoy better health than the general population. In occupational epidemiology, the absence of the healthy worker effect is a yellow warning sign for the presence of possible occupational risks. The sample observed also had a higher prevalence of obesity (11.3%, CI 95% 8.4%; 14.8%) than the general Italian population (7.5%) [76]. It was therefore appropriate to evaluate whether office workers were exposed to factors that promote overeating and obesity.

Occupational risk factors that could explain the increased risk in office workers include lifestyle, stress, sleep problems, and mental health disorders. In large cities, the lifestyles of office workers, who are generally forced to commute long distances, eat out, and limit time for exercise and relaxation, are certainly not conducive to maintaining well-being. Additional lustful habits could worsen the situation. However, the suspected cases of EDs in the offices we monitored did not differ in smoking and drinking habits from those of the other workers. This finding does not confirm reports of a possible association between EDs and recreational tobacco and alcohol consumption. The literature indicates that individuals with BED and AN are significantly more likely to be lifetime smokers than healthy controls [77]. A recent meta-regression observed that current smoking rates are significantly higher among individuals with EDs (especially women) than in the general population [78]. Smoking may function as a weight management strategy in these populations, highlighting the need for integrated smoking cessation interventions in the treatment of eating disorders. Alcohol abuse has also been reported frequently in association with EDs [79]. In adults with EDs who participated in the National Epidemiological Survey on Alcohol and Related Conditions III (NESARC-III), depression and alcohol abuse were the primary comorbidities [9]. In our cohort, depression played a significant role in the risk of EDs, while alcohol abuse did not appear to be associated. To explain this apparent divergence from the literature, we must consider that the case studies on which the previous claims are based consisted of individuals undergoing treatment for EDs and, in one case, also for alcohol addiction. On the contrary, the office workers we examined were undiagnosed and untreated, and the severity of their level of alcohol use was probably less than that quoted in the literature. An alternative explanation could be that the employees under-reported their alcohol consumption.

With regard to tobacco smoking, the data observed among our sample of office workers were worse than those found in a random sample of the general Italian population, in which the prevalence of current cigarette smoking is estimated at 23% among men and 19% among women [80]. Among our office workers, one-third were current smokers and one-fifth had quit or switched to e-cigarettes; less than half were non-smokers. The smoking rate in this sample was very high, even worse than that found in our country among healthcare workers, who are rightly criticized because they give excellent advice on smoking but do not follow it themselves. A 2025 meta-analysis reported that 22.9% of Italian doctors and 37.6% of nurses are smokers [81].

As regards physical activity during free time, the sample we observed had a worse lifestyle than the general Italian population, in which sedentary behavior during leisure time was observed in 34% of Italian men and 45% of women. The situation described here was worse because the lack of leisure time activities was added to the sedentary nature of work [80]. According to national estimates, the prevalence of a correct lifestyle, defined as non-smoking, regular physical activity, and adherence to at least five appropriate eating behaviors, is 7% for men and 12% for women [80]. Among our sample, the prevalence of healthy lifestyles was less than 2%. In conclusion, all the office workers presented worse lifestyles than the Italian population, but no significant differences emerged between suspected EDs and other workers. Probably, the high prevalence of unhealthy lifestyles in a numerically small sample masked any associations with EDs. The first hypothesis we made, that EDs were associated with unhealthy lifestyles, was neither confirmed nor rejected by this study. However, the low level of well-being of the monitored cohort calls for health promotion interventions.

In our study, eating disorders were slightly more frequent in women than in men, but no significant statistical difference was observed. Once again, the sample size prevented us from detecting a difference. In the United States, the estimated lifetime prevalence of EDs is around 1 in 5 females and 1 in 7 males at 40 years of age [82]. The gender gap is largest during adolescence but tends to narrow in middle age. Our findings are in line with a study conducted in a German population, indicating that EDs are slightly more prevalent in females than in males during adolescence; however, no differences are observed in adults [83].

Age did not reveal a significant relationship with EDs in this census. Our observations indicated that nearly all suspected cases of EDs were linked to binge eating and obesity. The prevalence of overnutrition disorders in this population with an average age of over 46 years is in line with reports indicating the onset during the peri- or post-menopausal period in women, or at a similar age, in men [84]. Obesity was very common among our employees, a fact that could have an adverse impact on productivity. In the United States, the economic and social burden of overnutrition disorders is substantial: in the fiscal year 2018–2019, the total cost was nearly $400 billion [85]. It is not easy to evaluate the impact of EDs because international estimates, such as the Global Burden of Disease, consider only AN and BN and neglect more frequent disorders such as BED and OSFED. Together, the latter cause 3.7 million (95% UI 2.0–6.5) DALYs globally [86]. A study involving more than 22,000 US adults from the National Health and Wellness Survey revealed that respondents with BED manifested markedly higher rates of absenteeism, presenteeism, work productivity loss, and activity impairment compared to those without BED [87]. Another study in the United States involving more than 110,000 workers found that obesity and BED were linked to the highest rates of absenteeism, presenteeism, and productivity impairment [88]. An interesting development of our study could include an analysis of sick leave in workers with BED and obesity. If companies perceived a productivity benefit, they would be encouraged to implement health promotion programs.

This study confirmed that poor sleep quality is associated with EDs (OR = 1.315, CI 95% 1.186; 1.457). Work can affect sleep quality in various ways, both by altering biorhythms and interfering with chronotype during shift work, and by hindering sleep onset and promoting wakefulness due to stress. Studies conducted on shift workers have failed to disentangle the two factors. For this reason, in order to evaluate the effect of sleep deprivation that is not related to work hours, we chose to investigate workers who do not rotate shifts. Our results confirmed the association between EDs and poor sleep quality, although the causes of this poor quality still need to be clarified. Many social and cultural factors, including sleep timing [89], exposure to media [90],[91], and devices that emit blue light [92], may disturb sleep. Office workers are an ideal group for assessing the impact that each of these different causes and other factors, such as family responsibilities [93],[94], financial difficulties [95], parenthood [96], and occupational stress [97],[98], can have on sleep quality.

Sleep deprivation can, however, also have clinical causes that may be undetected. One of these is obstructive sleep apnea (OSA), a serious and much underdiagnosed condition. More than half of the world's population (estimated combined prevalence 54%, CI 95% 46%–62%) is affected by different levels of severity of OSA [99]. An estimated 6%–17% share of the population could have an apnea-hypopnea index (AHI) ≥ 15 events/h, requiring immediate treatment. This percentage reaches 49% in an older population where cases of AHI > 5 could be as high as 90% in men and 78% in women [100]. Most people are unaware of being affected by this disease because of limited access to polysomnography, so very few cases are correctly diagnosed and treated [101]–[103]. Screening through questionnaires can induce many individuals to undergo the necessary tests and control this disease. The full form of the STOP-Bang questionnaire is a highly sensitive and specific tool for diagnosing OSA. A score ≥3 can detect moderate to severe OSA (AHI > 15) in 93% of cases and severe OSA (AHI > 30) in 100% of cases [104]. The two questions we used can reveal a significant proportion of cases, but inevitably miss those in which apnea or snoring cannot be confirmed because individuals sleep alone. In our survey, the workers who were aware of a sleep problem were certainly fewer than those who were affected by this morbid condition. Interestingly, however, suspected OSA cases were significantly associated with EDs.

The association between OSA and EDs that emerges from this study is not unexpected, given the link between EDs and metabolic disorders [105],[106] and the association between overeating disorders and obesity [107]–[111]. BED, NES, and other eating disorders are associated with OSA and other significant comorbidities such as depression, anxiety, and impairment of the mental quality of life [112]. Studies on obese patients awaiting bariatric surgery have shown that insomnia and depression in obese individuals are associated with eating habits [113], and that patients with OSA are very likely to have a diagnosis of previous BED and current major depression [114]. Considering the association of OSA with psychiatric disorders [115]–[117], the presence of EDs in association with OSA becomes an important and special topic [118]. Not only the presence of clinically evident ED but also disordered eating could promote OSA. Longitudinal data on over 145,000 individuals from the Nurses' Health Study (NHS) (2002–2012), NHS II (1995–2013), and Health Professionals Follow-up Study (1996–2012) demonstrated that better diet quality was associated with lower OSA risk, while proinflammatory diet-related mechanisms were important in the development of OSA [119]. EDs and OSA are two important and currently little-known diseases that are linked by various mechanisms. The study of the latter would probably improve treatment and the quality of life for patients.

In this study, a highly significant correlation was found between work-related stress and the risk of EDs (OR = 5.379, CI 95% 2.291; 12.631). Occupational stress may be linked to EDs, as these disorders may be utilized as a coping mechanism [120]. A complex association between EDs and occupational stress, particularly in obese workers, has been documented [121]. Job strain increases the probability of bipolar disorder in people with binge eating; in this relationship, body mass index acts as a moderating factor [122]. Occupational distress among UK doctors has been associated with an elevated risk of EDs, sleep disturbances, alcohol abuse, and drug use [79]; it has also been associated with high levels of EDs among nurses [123]. The ELSA-Brazil study identified a correlation between job strain and BED [124]. Furthermore, an inverse relationship exists between work engagement, characterized by a positive attitude toward one's job, and disordered eating among female workers [125]. On the other hand, work addiction, characterized by excessive commitment to work, has been linked to an increased risk of EDs [126]. Compulsive overworking correlated with EDs among Polish students [127]. Individual sensitivity to stress certainly plays a role in this relationship; workers with a high susceptibility to work-related stress manifested an increased risk of BED [128]. In the German LIFE-Adult-Study, excessive work demands, social overload, and the management of negative emotions were identified as predictors of food addiction [129].

Many studies have associated the onset of EDs with a prior experience of trauma [130]–[132]. In this study, we did not investigate trauma experienced by workers during their childhood; instead, we concentrated on studying recent events. The demonstration of an association seemed particularly significant because it revealed a phenomenon that had not been highlighted previously and which will certainly require further confirmation. Distress in workers with EDs may stem from factors other than traditional occupational stressors. One such factor is bullying. Weight stigma, defined as the marginalization and social devaluation of individuals based on larger body size, is linked to body image issues, psychological distress, disordered eating, and adverse health outcomes among racially and ethnically diverse young men, irrespective of their body size [133]. In this study, physical or verbal violence experienced at work was significantly associated with the risk of EDs (OR = 4.365, CI 95% 1.354; 14.067). Although it is known that office workers are less exposed to violence than other categories of workers [134], exposure to uncivil and violent behavior is a risk that should not be underestimated. Other common everyday life events were also significantly associated with EDs. Near-miss driving accidents (OR = 3.322, CI 95% 1.259; 8.764) and domestic injuries (OR = 3.895, CI 95% 1.042; 14.562) are distressing events that may disrupt the regular organization of family life and work/family balance and may therefore intervene to alter the eating pattern in an individual susceptible to EDs. Finally, the most serious traumas that occurred in the year preceding the medical examination had a highly significant association with the risk of EDs (OR = 4.471, CI 95% 1.990; 10.044, *p* < 0.001) and represented a significant predictor even in multivariate logistic regression (OR = 2.689, CI 95% 1.024; 7.061, *p* < 0.05), thus confirming the very relevant role of these events. Moreover, the literature indicates that negative avoidance and emotion-focused coping strategies may be significant factors in the genesis of EDs [135]. The relationship between negative events and EDs could be investigated more extensively through a longitudinal design that considers variation in the level of psychosocial stress and coping strategies.

In children and adolescents, the literature indicates an association between negative emotional states, such as anxiety and depressive feelings, and maladaptive eating behavior [136],[137]. Depression, anxiety, and general distress may be mediating factors between childhood trauma and the development of EDs [138]. The same mechanisms may also be operative in adults affected by common mental disorders. If we consider that life events affect all human beings equally, this association could indicate a tendency to react to stress by overeating. In the literature, it has been observed that this coping method is often associated with depression [139],[140]. In our cohort, both subclinical anxiety (OR = 1.409, CI 95% 1.227; 1.619) and depression (OR = 1.555, CI 95% 1.335; 1.811) were found to have a highly significant association with the risk of EDs, as we predicted in the fifth hypothesis, and depression was confirmed to be highly significantly associated with EDs also in the multivariate model (OR = 1.436, CI 95% 1.095; 1.883, *p* < 0.001).

This study has the merit of having investigated for the first time the prevalence of EDs in a population of office workers by examining the occupational and personal factors that are associated with EDs. By studying a population that does not work night shifts, we were able to demonstrate that sleep disruption rather than shift work is a risk factor. Furthermore, it was possible to evaluate for the first time the association between OSA and EDs without the confounding effect caused by night work. Our study also demonstrated that office workers generally adopt unhealthy lifestyles. These findings and the analysis of the impact that EDs have on the physical and mental health and productivity of office workers provide valid evidence for encouraging the implementation of health promotion campaigns. We hope that this will encourage companies to fund health promotion programs that, in addition to identifying EDs, also assess and prevent their metabolic and cardiovascular effects and those on mental health.

However, our study has several limitations. First of all, our observations are based on a small number of office workers; this probably prevented us from demonstrating the significance of some differences between suspected cases of EDs and other colleagues. Furthermore, the study design (a cross-sectional census) prevented us from identifying the direction of the associations observed. Nevertheless, the interpretation of our results is consistent with the literature. The ED-QS questionnaire, which was developed specifically for screening, provides suspicion of disease and refers to further specialist assessments for diagnosis. The lack of a clinical diagnosis is a major limitation in this study, although the main aim of the study was to identify workers with these disorders. None of the suspected EDs had previously received treatment. Suspected cases were invited to undergo tests at National Health Service facilities. The occupational physician would then have been able to verify the effects of these tests during subsequent routine medical examinations. Another limitation is our use of a convenience sample, which prevents the application of our findings to other office workers. However, there were no significant differences in occupational risks and in socioeconomic and cultural conditions between one office job and another. The combination of data collection with the medical examination performed in the workplace and the very high participation in the census leads us to believe that the collected data are reliable. We hope that further studies can be conducted with the same simple methodology to confirm the results achieved. Finally, by choosing very brief survey instruments (sometimes only a single question), we reduced the study's potential; however, this choice was inevitable in a survey of a large population since completion of the questionnaire must not reduce the time dedicated to medical examinations in the workplace that are always conducted within a tight deadline to avoid interfering with production needs.

This study has highlighted several elements that deserve clarification in future research, and it may serve to improve research tools. The relationship between different types of traumas and the onset of sleep or mental health problems may be mediated by stress. Since the occupational stress model we used is not the most suitable for assessing the consequences of a home injury, bereavement, or traffic accident, a different stress model with a broader construct could be used to report the sequence of events in a longitudinal study.

Another aspect that deserves further investigation concerns the impact of EDs on physical health. This unfunded study lacked blood glucose and lipid measurements that could have indicated a relationship between EDs and metabolic syndrome or cardiovascular risk. Considering the negative impact EDs have on productivity, companies should be encouraged to address this issue.

## 5. Conclusions

A significant proportion of office workers suffer from overeating and obesity. Unhealthy lifestyles contribute to eating disorders associated with trauma, sleep disturbances, stress, and emotional distress. Disordered eating has a negative impact on workers' physical and mental health and is likely to reduce the quality of life and productivity. Although eating disorders are not an occupational disease, it is important that the problem be addressed by worker health surveillance services, as various workplace factors may be associated with them. Better sleep hygiene, stress reduction, and emotional trauma counseling could contribute to promoting the health of office workers.

## Use of AI tools declaration

The authors declare they have not used Artificial Intelligence (AI) tools in the creation of this article.
